# WHEN LOVE HURTS: AMBIVALENT TIES INFLUENCE THE LINK BETWEEN FUNCTIONAL LIMITATIONS AND WELL-BEING

**DOI:** 10.1093/geroni/igac059.1219

**Published:** 2022-12-20

**Authors:** Misha Haghighat, Meng Huo

**Affiliations:** University of California, Davis, Davis, California, United States; University of California, Davis, DAVIS, California, United States

## Abstract

Functional limitations may co-occur with worse emotional well-being in older adulthood. Research has found that this association likely varies by social factors, with social support buffering well-being from physical declines, and strain exacerbating it. Yet, older adults’ functional limitations may contribute to more ambivalence in their social relationships, which involves both support and strain. Such ambivalence may exacerbate older adults’ emotional well-being in the face of physical limitations, but we know little about whether and how this occurs. The current study innovatively examined how older adults’ overall ambivalence moderates the association between functional limitations and depressive symptoms, and captured emotional mood among older adults with functional limitations when they had encounters with ambivalent partners throughout the day. Study participants (N = 313; ages 65+) were from the Daily Experiences and Well-being Study. Participants reported on functional limitations, depressive symptoms, and relationship quality with each social partner in a 2-hour in person interview, followed by reports of social encounters every 3-hours across 5-6 days. We observed a significant moderating effect of overall ambivalence on the association between functional limitations and depressive symptoms, such that this association was more salient among older adults with more ambivalent social networks. Further, only unpleasant encounters with ambivalent social partners, but not those with non-ambivalent social partners, compromised mood among older adults with functional limitations. These findings may advance our understanding of older adults’ ambivalent ties in the context of physical declines, and help identify therapies that may enhance well-being for older adults with health limitations.

